# Radiographic evaluation of posterior selective thoracolumbar or lumbar fusion for moderate Lenke 5C curves

**DOI:** 10.1007/s00402-016-2570-1

**Published:** 2016-09-21

**Authors:** Yanbin Zhang, Guanfeng Lin, Jianguo Zhang, Jianwei Guo, Shengru Wang, Yang Yang, Jianxiong Shen, Yipeng Wang

**Affiliations:** Department of Orthopedics, Peking Union Medical College Hospital, 1 Shuai Fu Yuan, Beijing, 100730 People’s Republic of China

**Keywords:** Selective fusion, Thoracolumbar or lumbar, Indication, Maximal correction, Disc wedging

## Abstract

**Introduction:**

Posterior selective thoracolumbar or lumbar (TL/L) fusion with pedicle screw constructs for adolescent idiopathic scoliosis (AIS) has been studied in a few researches. However, few studies have discussed the indication for selective TL/L fusion and the behaviors of its adjacent disc angle. The present study aims to discuss the indication for posterior selective TL/L fusion and the behavior of the adjacent disc angle.

**Methods:**

45 consecutive cases of AIS undergoing posterior selective TL/L fusion were retrospectively evaluated, with an average follow-up of 36 months. Radiographs were reviewed to determine the coronal curve magnitude and the sagittal alignment preoperatively, postoperatively and at final follow-up. Thoracic curves in groups A had a correction loss of more than 5°, while thoracic curves in group B had a correction loss of not more than 5°.

**Results:**

The coronal curve magnitude of the TL/L curve averaged 44° preoperatively and it was corrected to 6° immediately with a correction rate of 84.8 %. At final follow-up it was 9° with a correction loss of 3°. The minor thoracic curve was 26° preoperatively, and the convex side bending curve magnitude averaged 8° with a flexibility of 72.7 %. It was corrected to 13° immediately with a spontaneous correction of 48.5 %. At final follow-up it was 14° with a correction loss of 1°. UIVA decreased from 4° to 2° after surgery, and it was 2° at final follow-up. LIVA decreased from 7° to 4° after surgery, and it was 5° at final follow-up. Maximal correction of TL/L curves in group A is significantly less than that in group B. 1 patient received revision surgery to fuse the progressive thoracic curve.

**Conclusion:**

Posterior selective TL/L fusion with pedicle screw constructs allows for spontaneous thoracic correction and maintains coronal and sagittal balance during the follow-up. Maximal correction instead of undercorrection was recommended for moderate Lenke 5C curves. Disc wedging could be improved after surgery and well maintained during the follow-up.

## Introduction

The goal of corrective surgery in adolescent idiopathic scoliosis (AIS) is to achieve global spinal balance with optimal coronal and sagittal alignment and axial derotation, while sparing motion segments. Sparing motion segments and saving motion capability of the spine have becoming the most controversial part of surgical correction. Since the spread of selective thoracic fusion [1–3], selective thoracolumbar or lumbar (TL/L) fusion drew spine surgeons’ attention. There are a few studies on anterior or posterior selective TL/L fusion for Lenke 5C curves [4–6]. The possible advantages of anterior approach may include better visualization, the less demanding nature of the technique, less fusion segments and better inter-body fusion than posterior approach [4, 5, 7]. Issues with the anterior approach included instrumentation failure, pseudarthrosis, and a kyphogenic compression mechanism [8, 9]. With the wide application of the pedicle screw constructs, most spine surgeons preferred to perform posterior-only procedure with better curve correction, less loss of correction over time, and shorter hospital stays [5]. Posterior selective TL/L fusion is becoming widely accepted as a preferred treatment for AIS with structural TL/L curve and compensatory thoracic curve (Lenke 5C). We retrospectively investigated patients treated with posterior selective TL/L fusion to discuss the indication for selective TL/L fusion and radiographic features like the behavior of the adjacent disc angle.

## Materials and methods

After the approval of the institutional review board of the hospital, 45 patients of Lenke 5C AIS with TL/L curvature and minor thoracic curve were identified in a single institution for the time periods from January 2006 to December 2012, with an average follow-up of 36 months (range 24–105 months). Radiographs, clinical charts, and operative reports were reviewed. Criteria for Lenke 5C classification [10] were used, and confirmed with another independent physician examiner familiar with this classification. Specifically, a Lenke 5 curve can be defined as an idiopathic structural curve with the apex from the T12 body to the L4 body. The main thoracic and upper thoracic curves were nonstructural, which means that their magnitude is less than the primary structural curve, they bent out to be less than 25° on convex side bending radiographs, and no sagittal kyphosis criteria were met (T10–L2 and T2–T5 are less than 20°). Exclusion criteria were: age >20, non-idiopathic curve, follow-up <2 years, incomplete follow-up materials and poor radiographic images to measure.

### Surgical technique

The patient was placed prone on a radiolucent spinal frame after administering intubated general anesthesia. After surgical exposure, pedicle screws were placed with free hand technique. Once the screws were in place, intraosseous placement was confirmed via C-arm image intensifier. Posterior release were performed where was needed. The convex rod was placed first in all patients. Curve correction was achieved with direct apical vertebral body derotation (VBD), rod rotation and compression and/or distraction. Decortication of the posterior elements was performed and followed by bone graft finally. Sensory- and motor-evoked potentials were used intraoperatively.

### Radiographic parameters

Radiographic analysis included various parameters on the preoperative, immediate postoperative (within 2 weeks), and final follow-up radiographs. Curve magnitudes of thoracic and TL/L curves were measured on both long-standing AP films and supine side bending films. The radiograph measurements and analysis were performed by two individual investigators. We presumed the Cobb angle to be reliably measured to be within 5°. Flexibilities of both curves were calculated. The coronal global balance was defined as the horizontal distance between the C7 plumb line (C7PL) and the central sacral vertical line (CSVL). The Apical Vertebral Translation (AVT) was measured as the distance between the center of the apical vertebra and C7PL in thoracic curve or CSVL in TL/L curve. Lower instrumented vertebra (LIV) Tilt measured the inclination in degrees of the inferior endplate of the LIV to the horizontal plane. The horizontal plane was defined as the plane perpendicular to the long axis of the radiograph. The coronal lowest instrumented vertebra disc angle (LIVA) immediately below the LIV was measured as the angulation in degrees of the inferior endplate of the lower instrumented vertebra (LIV) relative to the superior endplate of the next caudal vertebra. The coronal upper instrumented vertebra disc angle (UIVA) immediately above the upper instrumented vertebra (UIV) was measured as the angulation in degrees of the upper endplate of the UIV relative to the lower endplate of upper adjacent vertebra. Sagittal curve magnitude was measured as follows: (1) T5–T12; (2) T10–L2; (3) L1–S1. UIVA and LIVA were also measured in the sagittal plane.

Potential errors may occur while measuring the angles on the radiograph (measurement error). This error has been investigated intensively and is suggested to be around ±5° [11, 12]. Most investigators have considered 5° of change or more to be clinically important [13–15], and in clinical setting, it is common for practitioners to make recommendations concerning treatment on the basis of an increase in the curve of 5° between two successive radiographs [12]. Based on above findings, if correction loss of the thoracic curve is more than 5°, we divided all cases into two groups. Thoracic curves in groups A had a correction loss of more than 5°, while thoracic curves in group B had a correction loss of not more than 5°. We compared the differences of immediate TL/L curve magnitude between two groups.

We calculated overall summary statistics in terms of means and SDs for continuous variables and frequencies for categorical. After the descriptive analysis, *p* value was calculated using independent sample *t* tests for continuous variables obeying normal distribution. For those not obeying normal distribution, non-parametric tests were used. We evaluated group differences for categorical variables using Chi-square or Fisher’s exact test. All analyses were performed using SPSS (version 17.0.0, Inc. Polar Engineering and Consulting).

## Results

**Fig. 1:**
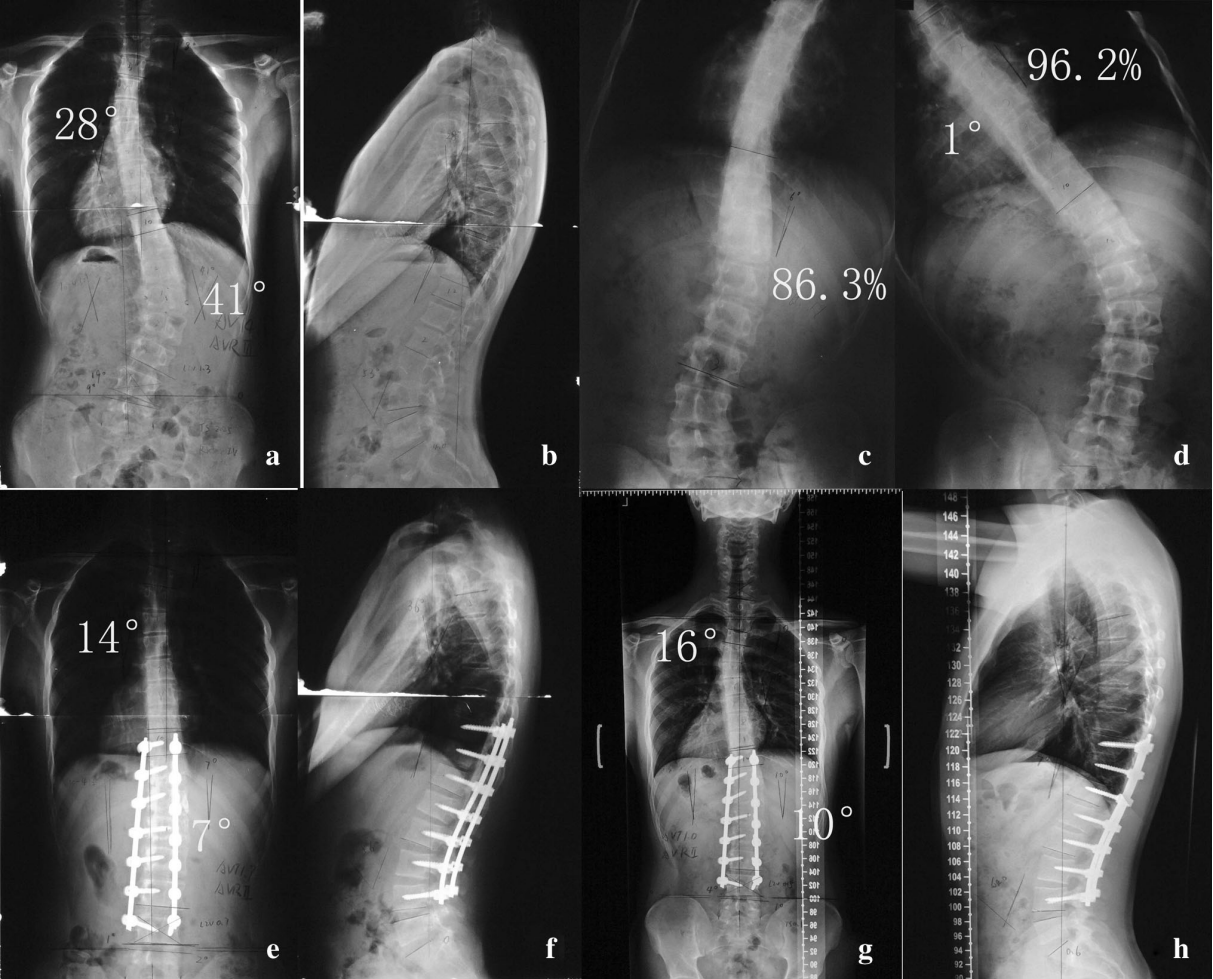
**a**–**h** A 14-year-old female patient. Flexibility of the thoracic and TL/L curve was calculated as 96.2 % and 86.3 %, respectively (**c**, **d**). After selective fusion, the thoracic curve spontaneously corrected from 28° (**a**, **b**) to 14° (**e**, **f**). At final follow-up, it was 16°, with a correction loss of 2° (**g**, **h**). Coronal and sagittal balances were well maintained both in the immediate post-operation and final follow-up

Patients list is shown in Table 1. The coronal curve magnitude of the TL/L curve averaged 44° (range 35°–72°) preoperatively and it was corrected to 6° (range 0°–22°) immediately with a correction rate of 84.8 % (range 47–100 %). At final follow-up it was 9° (range 0°–28°) with a correction loss of 3° (range 0°–14°). The minor thoracic curve was 26° (range 10°–43°) preoperatively, and the convex side bending curve magnitude averaged 8° (range 0°–18°) with a flexibility of 72.7 % (range 28–160 %). It was corrected to 13° (range 1°–30°) immediately with a spontaneous correction of 48.5 % (range 4.3–95.4 %). At final follow-up it was 14° (range 0°–32°) with a correction loss of 1° (range 0°–13°). Typical case is shown in Fig. 1. Coronal global balance was 21.3 mm (range 0–47 mm) preoperatively, 19.5 mm (range 0–43 mm) postoperatively and 10.9 mm (range 0–35 mm) at final follow-up. LIV tilt was corrected from 22° (range 10°–36°) to 4° (range 0°–18°), and at final follow-up it was 4° (range 0°–10°). UIVA decreased from 4° (range 0°–10°) to 2° (range 0°–7°) after surgery, and it was 2° (range 0°–8°) at final follow-up. LIVA decreased from 7° (range 0°–18°) to 4° (range 0°–8°) after surgery, and it was 5° (range 0°–18°) at final follow-up (Table 2).

**Table 1:** Patient characteristics and preoperative curve parameters

No.	Sex	Age	Follow-up	TL/L mag	Thoracic mag	Thoracic flexibility (%)	Lenke	Cobbs R (TL/L: T)
1	F	14	105	45	20	85.00	5C	2.25
2	F	20	100	30	20	95.00	5C	1.5
3	F	13	32	46	32	62.50	5C	1.43
4	F	12	78	36	28	100.00	5C	1.28
5	M	13	48	57	24	91.67	5C	2.34
6	F	13	45	44	30	90.00	5C	1.46
7	F	19	64	54	34	64.70	5C	1.58
8	F	15	30	35	24	62.50	5C	1.46
9	F	14	72	36	23	75.00	5C	1.56
10	F	14	72	41	30	53.33	5C	1.37
11	F	16	24	36	16	68.75	5C	2.25
12	F	15	60	49	32	62.50	5C	1.53
13	F	17	24	32	20	95.00	5C	2.25
14	F	13	29	56	22	54.54	5C	2.54
15	M	12	26	66	23	91.30	5C	2.87
16	F	13	38	28	10	160.00	5C	2.8
17	F	16	38	38	20	65.00	5C	1.9
18	F	19	28	42	22	77.27	5C	1.91
19	F	13	24	50	33	48.45	5C	1.51
20	F	14	24	45	24	87.50	5C	1.87
21	F	15	24	46	35	85.71	5C	1.31
22	F	14	30	51	33	60.60	5C	1.54
23	F	14	50	33	21	33.33	5C	1.57
24	F	16	24	40	28	35.71	5C	1.43
25	F	16	43	40	23	56.52	5C	1.74
26	F	14	24	36	25	36.00	5C	1.44
27	F	19	24	30	16	75.00	5C	1.87
28	F	13	32	51	43	57.50	5C	1.18
29	F	15	36	72	36	88.88	5C	2
30	F	15	24	46	25	28.00	5C	1.84
31	F	19	24	50	13	69.23	5C	3.85
32	F	17	24	34	22	77.25	5C	1.54
33	F	12	24	42	30	73.33	5C	1.4
34	M	16	24	69	40	86.04	5C	1.6
35	F	12	26	40	32	90.60	5C	1.25
36	F	14	26	58	39	64.10	5C	1.48
37	F	19	24	46	34	65.62	5C	1.35
38	M	16	24	41	26	96.15	5C	1.57
39	F	16	24	45	20	93.33	5C	2.25
40	F	15	24	29	20	80.00	5C	1.45
41	F	14	24	35	26	92.30	5C	1.35
42	F	13	24	35	13	76.92	5C	2.69
43	F	13	24	39	20	70.00	5C	1.95
44	F	15	24	50	32	43.75	5C	1.56
45	F	15	24	44	20	45.00	5C	2.2

*TL/L mag* thoracolumbar or lumbar curve magnitude, *thoracic mag* thoracic curve magnitude, *mag R (TL/L: T)* curve magnitude ratio of thoracolumbar or lumbar:thoracic

**Table 2:** Comparison of preoperative, postoperative and final follow-up coronal measurements

Parameter	Pre-op	Post-op	Follow-up	*p*
TL/L (°)	44 ± 7	6 ± 5	9 ± 5	<0.01*
Thoracic (°)	26 ± 7	13 ± 7	14 ± 8	<0.01*
GCB (mm)	21.3 ± 11.5	19.5 ± 13.3	10.9 ± 8.9	<0.01*
AVT (TL/L) (mm)	42.5 ± 12	12.9 ± 8.4	11.1 ± 8.8	<0.01*
AVT (T) (mm)	12.6 ± 6.8	15.2 ± 8.6	12.1 ± 9.1	0.681
LIV tilt (°)	22 ± 5	4 ± 4	4 ± 3	<0.01*
UIVA (°)	4 ± 3	2 ± 2	2 ± 2	<0.01*
LIVA (°)	7 ± 5	4 ± 3	5 ± 4	0.05

*TL/L* thoracolumbar or lumbar, *GCB* global coronal balance, *AVT* apical vertebral translation, *LIV* lower instrumented vertebra, *UIVA* coronal upper instrumented vertebra, *LIVA* coronal lower instrumented vertebra

* Means significant difference of the parameters between pre-op and final follow-up

For sagittal plane, curve magnitude from T5 to T12 was 18° (range 4°–45°) preoperatively, 24° (range 4°–36°) postoperatively and 28° (range 4°–58°) at final follow-up. Thoracolumbar junction (T10–L2) was 8° (range 0°–40°) preoperatively, 5° (range 0°–12°) postoperatively and 7° (range 0°–20°), and L1–S1 was 53° (range 29°–76°) preoperatively, 56° (range 28°–85°) postoperatively and 59° (range 43°–86°) at final follow-up (Table 3).

**Table 3:** Comparison of preoperative, postoperative and final follow-up sagittal measurements

parameter	Pre-op	Post-op	Follow-up	*p*
T5–T12 sagittal (°)	18 ± 9	24 ± 8	28 ± 12	<0.01*
T10–L2 sagittal (°)	8 ± 8	5 ± 4	7 ± 5	0.345
L1–S1 sagittal (°)	53 ± 12	56 ± 10	59 ± 10	0.038*
UIVA sagittal (°)	2 ± 3	1 ± 2	2 ± 3	0.319
LIVA sagittal (°)	13 ± 7	13 ± 6	14 ± 7	0.221
GSB (mm)	31.0 ± 23.5	35.8 ± 24.3	21.8 ± 16.8	0.017*

*GSB* global sagittal balance, *UIVA* sagittal upper instrumented vertebra, *LIVA* sagittal lower instrumented vertebra

* Means significant difference of the parameters between pre-op and final follow-up

We found that when maximal correction was defined as that the immediate TL/L curve magnitude was less than 10°, number of TL/L maximal correction in group A is significantly less than that in group B (*p* = 0.014) (Table 4).

**Table 4:** Comparison of maximal correction of TL/L curve between group A and B

	Immediate TL/L curve magnitude	
	≤10°	>10°
Correction loss of thoracic curve		
A > 5°	7	31
B ≤ 5°	5	2
Continuity correction *p* = 0.014		

### Complications and revision surgeries

Of all the 45 patients, only 1 patient received revision surgery to fuse the progressive thoracic curve. There was no neurologic complication or sign of pseudarthrosis on final follow-up radiographs.

## Discussion

Corrective surgery in adolescent idiopathic scoliosis (AIS) aims to achieve global spinal balance with optimal coronal and sagittal alignment and axial derotation. Selective TL/L fusion drew spine surgeons’ attention. Although anterior selective TL/L fusion has several advantages, most spine surgeons preferred to perform posterior-only procedures with the wide application of the pedicle screw constructs. Posterior selective TL/L fusion is becoming widely accepted as a preferred treatment for Lenke 5C curves (structural TL/L curve and compensatory thoracic curve).

Sanders et al. [16] suggested that the surgical success of selective anterior TL/L fusion depended on the structural changes in thoracic curve and the patient’s maturity. They stated that patients with closed triradiate cartilages, TL/L:T Cobb ratio more than 1.25 and thoracic curve magnitude on convex side bending film ≤25° would have satisfactory results. Oglivie et al. [17] stated the indications of selective TL/L fusion for double curves were the minor compensatory thoracic curve ≤40°, supple enough and no cosmetic deformity. Posterior procedure with pedicle screw constructs has powerful three-column corrective force and total different influence on the spine. Although there are several researches on posterior selective TL/L fusion with pedicle screw constructs, few of them have stated the indication for selective TL/L fusion. Lark et al. [18] found that Scoliosis Research Society Questionnaire scores and clinical balance are not significantly different between a matched set of patients that had either a selective or nonselective fusion of their Lenke 5 curve at 2 years postoperatively, but they did not discuss when selective TL/L fusion would be performed. Li et al. [19] suggested that patients with a preoperative thoracic curve >30° and a preoperative thoracic curve on bending >20° may not benefit from selective posterior fusion. In most conditions, when selective TL/L fusion would be performed depended on experiences of surgeons. In present study, out of 45 cases, the curve magnitude ratio of TL/L:T was more than 1.25 in 44 cases. The thoracic curve magnitude was not more than 40° except 1 case (43°) and all thoracic curve magnitudes on convex side bending films were less than 25°. Posterior selective TL/L fusion achieved 78.9 % correction for the TL/L curve and 44.7 % spontaneous correction for the minor thoracic curve immediately. At final follow-up, there is a correction loss of only 2.7° and 1°, respectively. Coronal balance was significantly improved and sagittal contours were well maintained with thoracic kyphosis increased a little but within normal range, at final follow-up (Tables 2, 3). It turned out to be an effective treatment for patients with Lenke 5C. In 1 case, the preoperative thoracic curve is 43°, its curve magnitude ratio of TL/L:T was 1.18, and thoracolumbar junction was 22°. Posterior selective TL/LT fusion was performed and the unfused thoracic curve was 21° at first erect. 30 months after surgery, the unfused thoracic curve progressed to 32° with cosmetic deformity. A revision surgery was indicated. Based on above data, we recommended thoracic curve magnitude ≤40°, convex bending curve magnitude <25° and curve magnitude ratio (TL/L:T) ≥1.25 as the indications for successful posterior selective TL/L fusion with pedicle screw constructs.

Undercorrection has been widely accepted in selective thoracic fusion. As stated by Von Lackum and Miller [20], it is desirable to achieve a correction of the primary thoracic curve that is not beyond the ability of the compensatory lumbar curve to balance the patient in selective thoracic fusion. When dealing with lumbar curves of a larger magnitude, the posterior approach, being capable of achieving strong corrective forces of the thoracic curve, is at risk of correcting the thoracic scoliosis beyond the capability of the lumbar curve to compensate and balance the spine in selective fusion for Lenke 1C [3, 21, 22]. Same perspectives had occurred in treatment for Lenke 5C curves. On one hand, for a balanced spine, complete correction of the instrumented curve was not suggested through anterior approach in Lenke 5C and a residual curve must be left to compensate the structural part of the thoracic curve [23]. But the definition of “residual” was not described. If the “residual” TL/L curve was too large, the spontaneous correction of thoracic curve would be incomplete and it may progress during follow-up. On the other hand, Huitema et al. [24] reported that spontaneous correction of the thoracic curve is a reflection of the TL/L curve correction in AIS in anterior selective TL/L fusion. It means, the more the TL/L curve was corrected, the more the thoracic curve can spontaneously correct itself. But if the TL/L curve was corrected so much that the thoracic curve failed to compensate, decompensation may occur. So it is maximal TL/L curve correction to achieve better spontaneous correction for thoracic curve, or undercorrection to gain a balanced spine? A Chi-square test showed us that if the immediate postoperative curve magnitude of the TL/L curve (ILCM) was not more than 10°, the correction loss of the thoracic curve during the follow-up would be not more than 5°. The difference is statistical significant (*p* = 0.014). So we recommend the residual TL/L curve should be not more than 10°, approximately maximal correction, when treating moderate Lenke 5C curves. But for Lenke 5C curves that did not comply with our indication, undercorrection may be needed if selective TL/L fusion were performed. In this condition, achieving a balanced spine instead of better correction would be main purpose.

Adjacent disc wedging is the radiographic characteristic after anterior selective TL/L fusion, and the coronal UIVA and LIVA increased significantly at final follow-up [7, 18, 25]. Less fusion segments [26, 27] and more excessive compression of the convex side [25] in anterior procedure may explain this. Disc wedging was also noted after posterior procedure. Stasikelis et al. [28] suggested that overcorrection of the upper lumbar curve might explain the increased disc angle. Yu et al. [29] reported that posterior TL/L fusion could provide a better disc wedging compared to the anterior approach but with a longer fusion range. They found no significant difference in the disc wedging before and after surgery, and also between immediate post-operation and final follow-up. In present study, after posterior selective TL/L fusion the immediate coronal UIVA and LIVA decreased significantly (LIVA: *p* = 0.003, UIVA: *p* = 0.001), which is different from previous studies. The difference between immediate post-operation and final follow-up was statistically non-significant (LIVA: *p* = 0.333, UIVA: *p* = 0.384). Adjacent disc angle decreased after surgery and could be well maintained during the follow-up. The mechanism by which the discs become wedged is poorly understood. Endplate calcification has been observed in discs of humans with scoliosis and in a porcine model of induced scoliosis, and is considered as a possible cause of nutritional compromise and consequent disc degeneration and wedging in scoliosis [30–32]. But we did not do examinations on endplate calcification for patients with AIS routinely. Stokes et al. [32] reported that reduced mobility was a major source of disc changes and may be a factor in disc deformity. In the previous literature, posterior procedure fused more motion segments and reduced more mobility compared with anterior procedure. However, improvement of disc wedging was noted after posterior selective TL/L fusion. There was a conflict with Stokes’ research. But their subjects were rat tails, which may be different from human spine. Moderate and flexible curves and better correction may explain behaviors of frontal UIVA and LIVA in the present study. Further investigations about the reason are still needed. In the process of rebalance, the changing of UIVA and LIVA are two major ways to remodeling the coronal alignment. They are the junctions of the grafted segment with the rest of the spine and will undergo considerable remodeling associated with re-equilibration of the whole spine after correction [23, 33]. Improvement and maintenance of LIVA and UIVA may mean better surgical outcomes and less re-equilibration.

Posterior selective thoracolumbar or lumbar fusion with pedicle screw constructs allows for spontaneous thoracic correction and maintains coronal and sagittal balance during the follow-up. Maximal correction instead of undercorrection was recommended for moderate Lenke 5C curves. Disc wedging could be improved after surgery and well maintained during the follow-up.
